# Economic and Performance Analysis of Achilles Tendon Rupture in the National Basketball Association

**DOI:** 10.1177/23259671241279388

**Published:** 2024-11-13

**Authors:** Kristopher Meadows, Fei Ye, Abram Qiu, Osasu Iyawe, Kenneth Kenneth-Nwosa

**Affiliations:** * The University of Texas Health Science Center at San Antonio, San Antonio, Texas, USA; Investigation performed at The University of Texas Health Science Center at San Antonio, Department of Orthopaedics

**Keywords:** basketball, ankle, Achilles tendon, cost of recovery, player performance

## Abstract

**Background:**

Achilles tendon ruptures are common and potentially career-ending injuries for National Basketball Association (NBA) players. Many studies have reviewed the impact of Achilles tendon ruptures on return to play (RTP) and performance, but there are no studies on their economic significance.

**Purpose/Hypothesis:**

This study aimed to analyze the economic and performance consequences of Achilles tendon ruptures usingthe cost of recovery (COR) for NBA franchises as well as preinjury salary/career success. It was hypothesized that players with higher preinjury salaries or performance would have an increased COR, higher rates of RTP, and more career success after their injury.

**Study Design:**

Descriptive epidemiology study.

**Methods:**

Publicly available data of NBA players who sustained an Achilles tendon rupture between 1990 and 2023 were analyzed. Data were retrospectively gathered by R software code to include players’ ages, positions, salaries, pre- and postinjury player efficiency rating, time missed after injury, and RTP. Performance impact was measured by advanced statistics: player efficiency rating, Win Shares per 48 Minutes, and Value Over Replacement Player. Two groups of 3 cohorts were created: All-Star, Starter, and Reserve versus group A (<$3,999,999), group B (≥$3,999,999 to ≤$8,999,999), and group C (>$8,999,999). Analysis of variance with post hoc Tukey tests for continuous data and Fisher exact tests for categorical data was used. Significance levels were set at *P* < .05.

**Results:**

A total of 37 players met the inclusion criteria and played between the years of 1992 through 2019. The mean COR that NBA teams faced was $4 million per player. The cumulative economic loss from Achilles tendon ruptures in the NBA between 1992 and 2019 was $117,578,851. Overall RTP was 78.38%, and 31.03% of players who returned to play were out of the NBA within 3 years. RTP to the highest playing level was highest in group B (45.5%), followed by group A (40%) then group C (12.5%). The COR of All-Star players, Starter players, and Reserve players averaged $5.7 million, $3.4 million, and $3 million, respectively.

**Conclusion:**

This study investigated the financial and performance implications of Achilles tendon ruptures among NBA athletes. Most players struggled to restore their preinjury performance, except for Reserve players. The findings provide valuable insights into the complexities of COR and postinjury performance.

Achilles tendon ruptures are devastating lower extremity injuries that affect many student and professional athletes. These injuries are typically seen in players of dynamic, high-impact sports such as basketball and volleyball, which frequently involve sudden plantarflexion of the feet. For an Achilles tendon rupture, the patient may wear a boot for up to 12 weeks, with restriction to only low-impact exercises for the first 6 months. High-impact exercises are added after this, and patients can then return to playing as soon as they feel comfortable. Treatments can range from nonoperative management consisting of casting and bracing to operative management, which entails either open repair or minimally invasive repair.^18^ Surgery involves restoring the proper length, function, and tensile strength of the tendon. Nonoperative treatments have shown a higher incidence of rerupture when compared with operative management of the injury.^15^

While many studies have focused on the performance of National Basketball Association (NBA) players postinjury, few have examined its economic significance on the league. This is important because a player's absence due to injury has a significant financial burden on NBA teams due to guaranteed contract agreements, which means that teams will have to continue to cover the full salaries of players who cannot physically contribute to games. By studying the cost of recovery (COR), more attention can be brought to the potential prevention of Achilles tendon ruptures and emphasize the need for effective treatments that will shorten players’ return to play (RTP) timeline. Our study examined the impact of Achilles tendon ruptures on players by comparing their preinjury and postinjury performances and the economic impact through the COR for NBA organizations that must fulfill player contracts. We hypothesized that players with greater preinjury performance and salary would have a greater COR, higher RTP rate, and more postinjury success.

## Methods

Code was written on R Version (4.2.3, Vienna, Austria) to scrape data from www.prosportransactions.com, a public, online database that tracks trades, draft picks, free agent signings, and injuries for each NBA team. This database has been used in previous orthopaedic studies regarding NBA athletes.^13^

**Figure 1.**
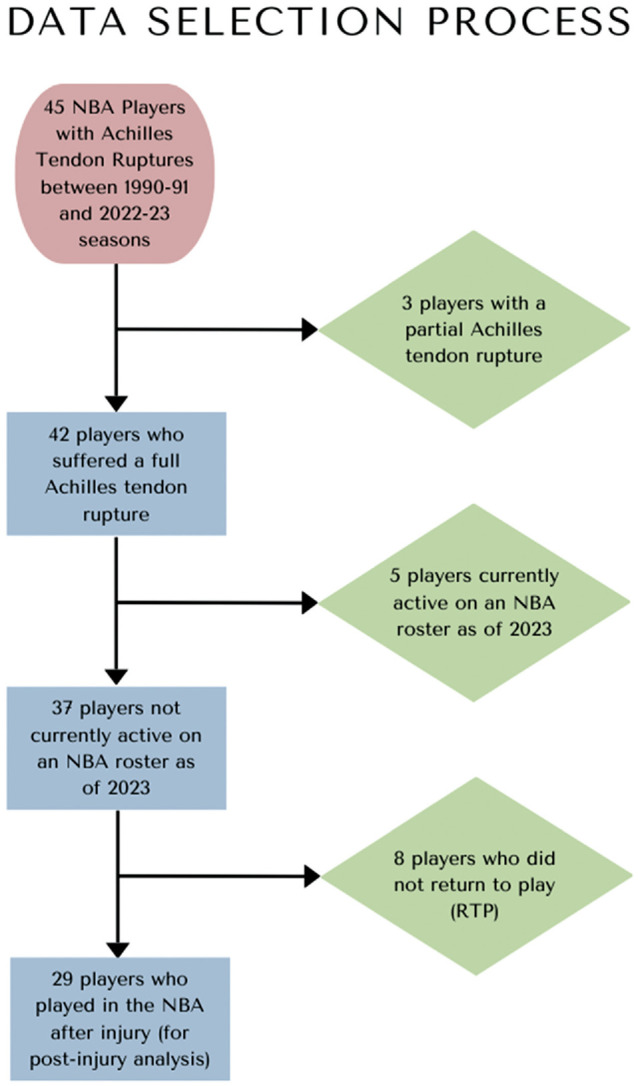
Flow diagram demonstrating how the final sample population was collected. NBA, National Basketball Association.

We reviewed NBA players with Achilles tendon ruptures between the 1990-1991 and 2022-2023 seasons. All data were obtained from publicly available sources, obviating the need for a formal institutional review process. Exclusion criteria encompassed NBA players who ruptured their Achilles tendon before the 1990-1991 season, players with only partial Achilles tendon tears, and players currently active on an NBA roster as of 2023. Players who sustained a career-ending injury were excluded from the postinjury analyses but included in RTP percentage and COR calculations. None of the players we found with documented Achilles tendon ruptures had documented tendon retears or required revision surgery. Figure 1 demonstrates our final cohort of population for analysis.

Salary information was obtained from HoopsHype (https://hoopshype.com/). Players were categorized into different groups based on their preinjury salaries: group A consisted of players who made <$3,999,999 per year, group B consisted of players who made between $3,999,999 and $8,999,999, and group C consisted of players who made >$8,999,999. Salary cutoffs for each group were based on dividing the total cohort into 3 groups with a roughly equal number of players; another study on professional athletes used similar groupings.^5^ Salaries for 3 years after injury were also obtained for postinjury and preinjury comparisons.

Players were also stratified into 3 groups based on their performance level: All-Star, Starter, and Reserve. This was based on a scale developed by a previous study, which graded NBA players based on their career accolades and starting lineup percentage.^21^ While this study has not been externally validated, it was included to provide another measure of player success, in addition to salary or statistical performance. The scale's 3 categories are shown in Table 1.

**Table 1:** Definitions of Career Success^*a*^

Level	Description
All-Star	Highly successful NBA player (first, second, or third team All-NBA, All-Star, Most Valuable Player, Defensive Player of the Year, or Sixth Man of the Year)
Starter	NBA player who started in >50% of games played
Reserve	Primarily a reserve NBA player who started in <50% of games played

a NBA, National Basketball Association.

The COR to the team was determined by multiplying the per-game salary of each player by the number of games the player missed due to the injury. It is important to note that this is a rough estimate, as it does not account for therapy-related costs such as surgery and physical therapy.

Player demographic data collected included age during injury, height (cm), weight (kg), and position played during injury season. Other injury-related data included the date of initial injury, number of games missed during the season of injury and the following season, total minutes played during the season of injury and the following season, date of the first game played postinjury, number of seasons played after the initial injury, RTP rate in the NBA, and retention rate in the NBA 3 years after injury. Details on surgical treatment were unable to be obtained. RTP was defined as playing ≥1 minute in an NBA game postinjury. We decided on this definition because other studies had used the same definition for RTP; for instance, Morse et al^14^ and Tramer et al^20^ used the same RTP definition in their analysis on NBA forearm/hand injuries and anterior cruciate ligament (ACL), respectively. Failing to RTP to the NBA also included leaving to play in an overseas league. RTP was ascertained based on whether the athlete played any documented minutes after his initial Achilles tendon rupture. Only regular season games were counted—neither preseason nor postseason games were calculated as part of games missed.

Player performance was evaluated with the player efficiency rating (PER) metric, which accounts for various positive (eg, free throws, assists) and negative contributions (eg, fouls, missed shots) in games and normalizes them per minute of play.^22^ This metric was chosen to represent performance because its per-minute basis prevents players who played limited minutes or games from biasing the data. Additionally, prior NBA-related studies have relied on PER as a trusted standard.^5,12,21^ The league mean PER is historically set at 15.^1^ PER was collected before and after injury and the difference was recorded. We also collected Value Over Replacement Player (VORP), which quantifies a player’s impact by measuring how much more valuable he is compared with a hypothetical replacement-level player. It encapsulates various aspects of a player’s performance, such as scoring, defense, and playmaking, into a single, interpretable number. This makes VORP a powerful tool for evaluating a player’s overall worth to a team, as it reflects the player's ability to outperform a readily available substitute.^8^ Additionally, our analysis included Win Shares per 48 Minutes, which calculates the number of wins a player contributes per 48 minutes of play. It offers a means of assessing a player’s contribution to a team's success by considering a player’s scoring, rebounding, assists, and defense during his playing time.^23^

Statistical analysis was performed with the aforementioned R software package, and continuous data were analyzed using analysis of variance with post hoc Tukey test. Fisher exact test was used to analyze categorical data, such as assessing the percentage of players who left the NBA 3 years after RTP or the percentage of players who returned to their highest playing level. Significance level was set at *P* < .05 for all tests.

## Results

In total, 45 NBA players sustained an Achilles tendon tear between 1990 and 2023. Of these players, 3 were excluded for a partially torn tendon, and 5 were excluded due to being currently active on an NBA roster as of 2023. A total of 37 players who met the inclusion criteria were further analyzed. Eight out of the 37 players sustained an NBA career-ending injury—meaning they did not play any minutes in the NBA after injury—and therefore were not included in the analysis concerning postinjury statistics. Thus, 29 players were used for the postinjury analysis portion of the study. The selection process is shown in Figure 1.

Out of the players who met the inclusion criteria, the RTP rate was 78.38% (29/37) and the return to the previous level of success was 27.03% (10/37). Only 54.05% (20/37) of players remained in the NBA 3 years postinjury. The mean age at the time of injury was 28.07 years, and the mean body mass index was 25.32. Mean height and weight were 2.02 m and 103.6 kg, respectively. The most Achilles tendon ruptures (n = 3) occurred in 2019. Of the given 37 players, 55.55% of these players were drafted in the first round, 28.88% were drafted in the second round, 15.55% were initially undrafted, and 59.52% were a top 15 draft pick.

Cumulative economic loss to NBA franchises was calculated from the sum of all players’ guaranteed salaries paid while they were not able to play in a season. The players in the All-Star group had a mean of $5.7 million with a sum of $51.7 million, the Starter players had a mean of $3.4 million with a sum of $41.2 million, and the Reserves had a mean of $3 million with a sum of $24.6 million.

The All-Star group played a larger number of minutes (23,901 minutes) before sustaining an Achilles tendon rupture compared with the other groups (Table 2). The All-Star group also exhibited a decline of 18,838 minutes played after injury, which is greater than that of Starter players (difference of 12,920 minutes when compared to the decline experienced by the All-Star group; *P* = .0093) and Reserves (difference of 13,770 minutes when compared to the decline experienced by the All-Star group; *P* = .012).

**Table 2:** Total Achilles Tendon Tears, Minutes Played, and Games Played for NBA-Injured Players at Each Preinjury Career Success Level^*a*^

	All-Star	Starter	Reserve
Total Achilles tendon tears, n	9	12	8
Seasons played after injury	4.4 ± 2.4	4.5 ± 2.3	2.4 ± 1.7
Minutes			
Minutes played before injury, total	**23,901.2 ± 9854.9**	10,837.5 ± 8014.0	6496.6 ± 4927.1
Minutes played after injury, total	5063.4 ± 4666.9	4919.3 ± 3282.6	1429.0 ± 1697.5
Difference in minutes played after injury	**18,837.8 ± 11,032.5**	5918.2 ± 9331.7	5067.6 ± 5609.5
Minutes played per game before injury, mean	**33.2 ± 3.9** ^*b*^	**27.8 ± 5.5** ^*c*^	17.9 ± 5.3
Minutes played per game after injury, mean	**22.1 ± 7.2** ^*b*^	**19.6 ± 3.4** ^*c*^	13.8 ± 2.3
Difference in minutes played per game after injury, mean	**11.1 ± 4.0** ^*b*^	8.2 ± 5.3	4.1 ± 6.3
Games played			
Games started before injury	**609.7 ± 265.5**	290.0 ± 208.5	75.9 ± 55.3
Games started after injury	**119.1 ± 114.9** ^*b*^	63.7 ± 58.9	8.1 ± 10.7
Difference in games started after injury	**–490.6 ± 275.1**	−226.3 ± 230.6	−67.8 ± 57.1
Games started before injury, %	**84.5** ^*b*^	**76.8** ^*c*^	23.4
Games started after injury, %	**54.3**	24.2	12.5

a Data are reported as mean ± SD unless otherwise indicated. Bold values indicate statistically significant difference among groups (*P* < .05). NBA, National Basketball Association.

b Indicates difference between groups for All-Star players and Reserve players only.

c Indicates difference between groups for Starter players and Reserve players only.

After the injury, the All-Star and Starter groups continued to play more, playing +8.3 minutes (*P* = .003) and +5.8 minutes (*P* = .030) more than the Reserve players, respectively, despite a larger reduction in the mean number of minutes played by the All-Star group (–11.1 minutes; *P* = .032) and the Starter group (–8.2 minutes; *P* = .237) (Table 2). The All-Star group also exhibited the greatest decline in the difference between the number of games started before and after the injury when compared with both the Starter players (difference of 264.3; *P* = .027) and the Reserve players (difference of 422.8; *P* = .001).

All Star–caliber players had a higher preinjury PER, 6.79 points greater than that of Reserve-level players (*P* = .003) (Table 3). However, in their first year playing postinjury, none of the groups exhibited a statistically significant difference in PER when compared with each other. Notably, there were also no statistically significant differences noted between postinjury and preinjury levels of PER. Reserve (*P* < .001) and All-Star players (*P* = .002) were more likely to return to their highest playing level after injury compared with Starter players.

**Table 3:** Mean Performance Data, Percentage Returning to NBA, and Percentage Recovered to Prior Playing Levels for NBA-Injured Players at Each Preinjury Career Success Level^*a*^

	All-Star	Starter	Reserve
Total Achilles tendon tears, n	9	12	8
Player statistics			
Age, y	**30.78 ± 2.68** ^*b*^	26.67 ± 2.90	27.12 ± 3.72
WS48 of season before injury	0.20 ± 0.23	0.09 ± 0.04	0.06 ± 0.05
WS48 of season after injury	0.13 ± 0.15	0.05 ± 0.05	0.06 ± 0.05
WS48 difference	0.07 ± 0.14	0.05 ± 0.06	0.00 ± 0.06
VORP of season before injury	1.51 ± 1.69	0.78 ± 0.90	0.16 ± 0.63
VORP of season after injury	0.81 ± 1.58	−0.25 ± 0.56	−0.17 ± 0.44
VORP difference	0.70 ± 2.02	1.03 ± 0.88	0.34 ± 0.70
PER of season before injury	**18.26 ± 4.00** ^*c*^	15.53 ± 2.32	11.47 ± 5.05
PER of season after injury	15.00 ± 4.82	11.85 ± 3.31	10.70 ± 3.20
PER difference	−3.26 ± 4.22	−3.67 ± 2.67	−0.78 ± 5.09
Return to play			
Out of NBA within 3 years, %	11.1	25.0	62.5
Returned to highest playing level, %	**22.2** ^*b*^	0.0	**100.0** ^*d*^

a Data are reported as mean ± SD unless otherwise indicated. Bold values indicate statistically significant difference among groups (*P* < .05). NBA, National Basketball Association; PER, player efficiency rating; VORP, Value Over Replacement Player; WS48, Win Shares per 48 Minutes.

b Indicates difference between groups for All-Star players and Starter players only.

c Indicates difference between groups for All-Star players and Reserve players only.

d Indicates difference between groups for Starter players and Reserve players only.

As expected, All-Star players made a higher salary during their injured season ($14,330,666; *P* = .01) (Table 4). They had the shortest recovery time at 267.6 days, followed by Reserve players at 340.0 days and Starter players at 367.1 days.

**Table 4:** Mean Salary Data, Length of Recovery Time, and Cost of Recovery PerNBA-Injured Player at Each Preinjury Career Success Level^*a*^

	All-Star	Starter	Reserve
Total Achilles tendon tears, n	9	12	8
Salary			
Preinjury, year of injury	**$14,330,666 ± $10,484,387**	$5,701,318 ± $4,093,261	$4,078,993 ± $3,102,403
Per game, year of injury	**$174,764 ± $127,858**	$69,528 ± $49,918	$49,744 ± $37,834
Postinjury year 1	**$14,506,558 ± $10,000,502**	$6,461,369 ± $3,439,027	$4,023,010 ± $3,153,149
Postinjury year 2	**$12,376,730 ± $8,252,464**	$4,839,959 ± $4,011,840	$3,841,313 ± $3,337,519
Postinjury year 3	$10,446,739 ± $10,357,007	$5,553,538 ± $5,526,133	$3,287,575 ± $2,789,321
Change, %			
Year 1	1.2	13.3	−1.4
Year 2	−15	−25	−5
Year 3	−15.6	14.7	−14.4
Games missed			
Season of injury	38.3 ± 23.1	50.4 ± 26.9	63.1 ± 25.5
Season after injury	41.7 ± 26.5	29.6 ± 18.7	36.6 ± 22.1
Total	80.0 ± 30.7	80.0 ± 36.9	99.8 ± 31.5
Recovery time, d	267.6 ± 81.0	367.1 ± 110.3	340.0 ± 79.9
Cost of recovery	$5,746,468	$3,438,299	$3,075,132

a Data are reported as mean ± SD unless otherwise indicated. Bold values indicate statistically significant difference among groups (*P* < .05). All values are in US dollars adjusted for inflation. The year of currency calculation was 2023. NBA, National Basketball Association.

Group C played more total minutes (21,899 minutes; *P* = .004) than group A (6643 minutes) before sustaining an Achilles tendon rupture (Figure 5). Furthermore, group C exhibited a steep decline of 17,080 minutes played from pre- to postinjury, greater than that of group A (difference in decline between the groups of −14,366 minutes; *P* = .009). Group C also averaged more minutes per game (33.3 minutes; *P* = .002) before injury than group A (21.8 minutes). Notably, there were no statistical differences in the number of minutes or mean minutes played postinjury; however, group C displayed a greater reduction in mean minutes played after injury compared with group A (difference of −7.1 minutes; *P* = .018).

Group C (*P* = .006) and group B (*P* = .04) both played more games before injury than group A; however, no statistical difference was noted after injury (Figure 5). Thus, both groups C (difference of −413.5 games; *P* = .03) and B (difference of −359 games; *P* = .04) saw steep declines in the difference in games played after injury compared with group A. Players in group C also started in more games preinjury compared with group A (552.6 games; *P* = .01), but the same correlation was not seen postinjury; thus, group C saw a statistically significant reduction in the difference in games started after injury (difference of −325.5 games; *P* = .02).

We also observed a greater PER for the season before the injury between group C and both groups A (+5.15; *P* = .03) and B (+5.51; *P* =.01) (Table 6). However, among the groups, there were no statistically significant differences found in the percentage of players out of the NBA within 3 years or the percentage of players who returned to their highest playing level. There were also no statistically significant differences in VORP or Win Shares per 48 minutes among the groups before or after injury.

**Table 5:** Total Achilles Tendon Tears, Minutes Played, and Games Played for NBA-Injured Players in Each Salary Group^*a*,*b*^

	Group A	Group B	Group C
Total Achilles tendon tears, n	10	11	8
Seasons played after injury	4.5 ± 2.4	3.0 ± 2.2	4.4 ± 2.3
Minutes			
Minutes played before injury, total	6642.7 ± 9407.4	14,138.0 ± 7063.1	**21,898.6 ± 10,488.8** ^*d*^
Minutes played after injury, total	3928.2 ± 2987.1	3473.2 ± 4147.5	4818.5 ± 4231.7
Difference in minutes played after injury	−2714.5 ± 10,836.8	−10,664.8 ± 6544.7	**–17,080.1 ± 10,872.4** ^*d*^
Minutes played per game before injury, mean	21.8 ± 7.5	26.4 ± 6.6	**33.3 ± 4.0** ^*d*^
Minutes played per game after injury, mean	16.8 ± 2.7	18.9 ± 6.8	21.2 ± 6.3
Difference in minutes per game played after injury, mean	−5.0 ± 7.5	−7.5 ± 3.1	**–12.1 ± 3.5** ^*d*^
Games played			
Games played in before injury	250.0 ± 286.1	**529.5 ± 198.3** ^*c*^	**647.4 ± 260.3** ^*d*^
Games played in after injury	227.1 ± 168.0	147.6 ± 138.0	211.0 ± 165.9
Difference in games played in after injury	−22.9 ± 381.6	**–381.9 ± 247.8** ^*c*^	**–436.4 ± 327.8** ^*d*^
Games started before injury	181.3 ± 266.2	303.6 ± 235.3	**552.6 ± 263.7** ^*d*^
Games started after injury	51.1 ± 53.8	55.9 ± 89.8	96.9 ± 106.3
Difference in games started after injury	−130.2 ± 272.5	−247.7 ± 194.2	**–455.7 ± 261.4** ^*d*^
Games started before injury, %	57.1	56.1	85.2
Games started after injury, %	27.3	23.9	42.8

a Data are reported as mean ± SD unless otherwise indicated. Bold values indicate statistically significant difference among groups (*P* < .05). All values are in US dollars adjusted for inflation. The year of currency calculation was 2023. NBA, National Basketball Association.

b Group A (<$3.9 million per year), group B ($3.9 million–$8.9 million per year), group C (>$8.9 million per year).

c Indicates difference between groups A and B only.

d Indicates difference between groups A and C only.

**Table 6:** Mean Performance Data, Percentage Returning to NBA, and Percentage Recovered to Prior Playing Levels for NBA-Injured Players in Each Salary Group^*a*,*b*^

	Group A	Group B	Group C
Total Achilles tendon tears, n	10	11	8
Player statistics			
Age, y	25.40 ± 3.72	**29.55 ± 2.34** ^*c*^	**29.38 ± 2.83** ^*d*^
WS48 of season before injury	0.08 ± 0.06	0.08 ± 0.05	0.21 ± 0.24
WS48 of season after injury	0.07 ± 0.04	0.05 ± 0.07	0.12 ± 0.16
WS48 difference	0.01 ± 0.05	0.03 ± 0.07	0.09 ± 0.14
VORP of season before injury	0.30 ± 0.75	0.79 ± 1.03	1.57 ± 1.66
VORP of season after injury	−0.34 ± 0.47	0.33 ± 1.58	0.34 ± 0.46
VORP difference	0.64 ± 0.67	0.46 ± 1.36	1.23 ± 1.75
PER of season before injury	13.97 ± 3.41	13.61 ± 4.96	**19.12 ± 2.54**
PER of season after injury	11.61 ± 2.22	11.82 ± 5.32	14.59 ± 3.59
PER difference	−2.36 ± 3.24	−1.79 ± 4.60	−4.53 ± 3.84
Return to play			
Out of NBA within 3 years, %	20.0	54.5	12.5
Returned to highest playing level, %	40.0	45.5	12.5

a Data are reported as mean ± SD unless otherwise indicated. Bold values indicate statistically significant difference among groups (*P* < 0.05). All values are in US dollars adjusted for inflation. The year of currency calculation was 2023. NBA, National Basketball Association; PER, player efficiency rating; VORP, Value Over Replacement Player; WS48, Win Shares per 48 Minutes.

b Group A (<$3.9 million per year), group B ($3.9 million–$8.9million per year), group C (>$8.9 million per year).

c Indicates difference between groups A and B only.

d Indicates difference between groups A and C only.

The preinjury, first-year postinjury, and second-year postinjury salaries of group C were greater than those of both groups A (*P* < .001) and B (*P* < .001) (Table 7). We found no statistically significant differences in pre- or postinjury salaries between group B and group C. Interestingly, although not statistically significant, only the mean salaries for group C decreased consistently throughout the postinjury years. Group A exhibited a remarkable 114% increase in their first-year postinjury mean salary while group B also witnessed a slight 5% increase. Both groups A and B also demonstrated a modest increase in third-year postinjury mean salaries compared with those of second-year postinjury. Furthermore, the COR of group C was more than that of group A (*P* = .004). We also found no statistically significant differences in the number of games missed among the groups during the season and after the season of injury.

**Table 7:** Mean Salary Data, Length of Recovery Time, and Cost of Recovery PerNBA-Injured Player in Each Salary Group^*a*,*b*^

	Group A	Group B	Group C
Total Achilles tendon tears, n	10	11	8
Salary			
Preinjury, year of injury	$2,296,886 ± $1,150,481	$6,072,135 ± $1,471,949	**$17,532,676 ± $8,875,818**
Per game, year of injury	$28,011 ± $14,030	$74,050 ± $17,951	**$213,813 ± $108,242**
Postinjury year 1	$4,915,884 ± $4,904,642	$6,372,644 ± $2,646,215	**$15,127,700 ± $10,131,455**
Postinjury year 2	$4,174,815 ± $3,372,428	$4,297,146 ± $3,275,298	**$13,928,793 ± $7,877,486**
Postinjury year 3	$4,539,460 ± $3,622,633	$4,734,087 ± $2,944,955	$11,918,910 ± $11,517,556
Change, %			
Year 1	114	5	−14
Year 2	−15.1	−32.6	−7.9
Year 3	8.7	10.2	−14.4
Games missed			
Season of injury	60.6 ± 27.2	49.5 ± 25.7	38.0 ± 23.5
Season after injury	29.3 ± 21.3	35.6 ± 21.0	42.2 ± 25.0
Total	89.9 ± 28.3	85.1 ± 44.3	80.2 ± 25.1
Recovery time, d	352.3 ± 73.7	350.7 ± 119.6	269.0 ± 86.3
Cost of recovery	$1,585,426	$3,830,191	**$7,449,061**

a Data are reported as mean ± SD unless otherwise indicated. Bold values indicate statistically significant difference among groups (*P* < .05). All values are in US dollars adjusted for inflation. The year of currency calculation was 2023. NBA, National Basketball Association.

b Group A (<$3.9 million per year), group B ($3.9 million–$8.9 million per year), group C (>$8.9 million per year).

## Discussion

Our key findings reveal a concerning scenario: all player achievement groups experienced a decline in playing time and statistical performance after injury. While All Star–caliber players had a higher preinjury PER (*P* = .003) compared with Reserve players, none of the player groups exhibited a statistically significant difference in PER when compared with each other in their first year playing postinjury. Interestingly, higher preinjury salaries persisted after the injury; postinjury performance declines did not significantly affect overall salaries across groups A through C. However, Achilles tendon ruptures were career ending for a significant portion (22%) of players, with Reserve players having the highest RTP rate but all player achievement groups experiencing career interruptions.

Many studies have focused on the RTP and performance of players after common orthopaedic injuries; however, the financial costs of injuries and their relationship with performance are often overlooked.^5,15,18^ To our knowledge, there has only been 1 similar published study, which focused on the economic impact of ACL tears on NBA players.^19^ Our study aimed to analyze relationships between the players’ COR with their performance and salary pre- and postinjury. We hypothesized that players with higher preinjury salaries or performance would have an increased COR, higher rates of RTP, and more career success postinjury. We found that while the previously higher-paid and -performing players had the greatest COR, they did not have greater rates of RTP or career success compared with the other players.

### All-Star, Starter, and Reserve Groups

Our analysis based on achievement groups demonstrates the extensive consequences for players who sustain a complete Achilles tendon rupture. For 22% (8/37) of the included NBA player cohort, Achilles tendon rupture proved to be a career-ending injury. For the remaining 78.4% (29/37), when comparing pre- and postinjury statistics, there was a notable decline in the number of in-game minutes played after injury, particularly for the All-Star players. Among all 3 groups, All-Star players also had the steepest drop-off in the difference in number of games started after their injury. These results were independent of career longevity, which was without statistically significant differences among the groups. Overall, teams utilized their players less frequently postinjury. However, playing time after injury still correlated with players’ preinjury success level.

This is interesting when considering the findings of player preinjury PER, as the All-Star group had a higher PER compared with the Starter group and a statistically significantly higher PER compared with the Reserve group. However, there was not a statistically significant difference in PER among the groups postinjury. This disproves part of our initial hypothesis that higher-performing players would continue to have more career success compared with others after their injury. Before their injuries, both the All-Star and the Starter groups started statistically significantly more games compared with the Reserve players. After their injury, All-Star players still started statistically significantly more games compared with the Reserve players, but our analysis revealed no statistically significant difference in the number of games started between the Starter and Reserve groups. This is important because All-Star players get paid more but teams will get disproportionately less performance from them compared with lower-ranked players after their injury. Moreover, teams may realize the discrepancy and tailor salaries in subsequent contracts to reduce the financial impact.

Despite the demonstration by our data of a nonsignificant difference in PER among groups postinjury, the All-Star players and Starter players still received significantly more playing time on average, 8.38 minutes (*P* = .003) and 5.84 minutes (*P* = .030), respectively. This may be explained by other intangible forms of value, such as intrinsic skill and veteran experience that can compensate for their physical decline, in-depth knowledge of a team's playbook, popularity in the league, and chemistry with the team. Thus, an NBA player's career standing can improve his longevity. This can be seen in the RTP trends in these groups, while not statistically significant. Only 11% of All-Star players were out of the league in 3 years compared with 25% of Starter players and 63% of Reserve players. This is supported by similar situations in other professional sports organizations. For example, ACL injuries in the National Football League (NFL), where Secrist et al^17^ found that players with initial annual earnings below the $2 million threshold tend to exhibit both lower mean salaries and a reduced likelihood of maintaining their presence within the league when compared with their uninjured counterparts. Conversely, individuals commencing their careers with earnings exceeding $2 million annually do not encounter a statistically significant salary reduction or reduced career duration. These findings underscore the significance of a player’s preinjury standing in shaping the extent of career impacts from injury.

Of note, we observed a statistically significant difference in the return to the highest playing level among the groups: 100% of Reserve players, 22% of All-Star players, and 0% of Starter players returned to their peak success level. These proportions invalidate part of our initial hypothesis that the group of higher-performing players would have greater career success compared with lower-performing players postinjury. This may be because the All-Star players and Starter players began at a higher level of play compared with the Reserve players, which increased the difficulty of making a complete return to their peak performance level. LaPrade et al^11^ similarly found that NBA players with Achilles tendon ruptures performed inferiorly to both themselves preinjury and uninjured players, who served as controls in their study.

### A, B, and C Salary Groups

Analyzing our cohort of players through an economic lens, we found that group C (>$8,999,999) had a statistically significant greater preinjury, first-year postinjury, and second-year postinjury salary than both group A (<$3,999,999) and group B (≥$3,999,999 to ≤$8,999,999). Notably, group A players’ salaries increased 114% at 1-year postinjury and remained greater than their preinjury salary in the second and third years after injury. On the other hand, while group B and C players’ salaries decreased in the second and third years after their injury, group B initially had a 5% increase in salary in their first-year postinjury. This suggests there may be an unknown effect modifier in our data since an injury would be expected to devalue a player, at least initially. For example, Secrist et al^17^ found that NFL players who sustained an ACL injury earned a mean of $2,070,521 less than matched controls over 4 postinjury years. Sigler et al^19^ also found that player performance, which is affected by injuries, correlates with how much they are paid.^2^ This supports our assumption that player salary differences at the population level are related to performance and any individual salary differences are not relevant. Additionally, the COR of group C was statistically significantly higher than that of group A, despite not missing any more games than group A did after injury. COR primarily impacts the NBA franchises that must pay the salary that is owed to their injured players. These differences help validate the salary groupings created and partially support our initial hypothesis that players with higher preinjury salaries would have an increased COR.

Vaudreuil et al^21^ also found that the highest-paid players with ACL injuries had a statistically significantly greater COR than the lowest-paid players with the same injury. Moreover, we calculated that the mean COR of Achilles tendon injuries was $4 million per player, which is also greater than the $2.9 million per player Vaudreuil et al calculated for ACL injuries. This shows that ankle injuries are a costlier injury for NBA franchises and highlights the importance of our study's findings about player performance pre- and postinjury.

Players in group C played statistically significantly more minutes, averaged higher minutes per game, and had higher PER before injury compared to group A, as shown in Table 5. However, group C's postinjury usage in total games played, minutes averaged per game, number of games, and PER decreased to levels that were not statistically significant different from either group B or group A. There were no statistically significant differences in Win Shares per 48 minutes or VORP among the groups before or after injury either, which stands out given the statistically significantly higher salary made by group C players before and after injury. There was also no statistically significant difference in the rates of retiring from the NBA within 3 years among the groups; furthermore, there was no statistically significant difference in the groups’ ability to return to former performance. These results disprove part of our hypothesis that higher-paid players would continue to have more career success compared with others.

This discrepancy in salaries and postinjury utility could be interpreted in different ways. To start, advanced statistics may not always provide a fully accurate reflection of player performance given the inherent complexity of the game. Additionally, players may provide other intangible assets that transcend advanced metrics and box scores. Adler developed an economic model to show that considerable differences in earnings could arise even without differences in talent or ability among performers.^3^ The study points to a performer's popularity, or public recognition as a star, as a reason for observed differences in stardom. Humphreys and Johnson^9^ expanded on Adler’s^3^ work and concluded that a player's popularity, as well as talent, can influence attendance at NBA games. Thus, a player's popularity can drive ticket sales and generate additional revenue for the franchise.

Investigating this issue through a financial lens provides valuable and unique insights into the challenges that NBA teams face because of Achilles tendon rupture injuries. This information could then be used to develop strategies to reduce the financial impact of Achilles tendon rupture on NBA teams. For example, franchises can determine how much to invest in more comprehensive injury prevention programs. The current NBA Collective Bargaining Agreement, a contract between the NBA and the National Basketball Players Association, began on July 1, 2023, and runs through the 2029-2030 NBA season.^6^ Although the current Collective Bargaining Agreement limits the salary cap increase to just 10%, projections indicate that players could exceed an $80 million base salary as soon as 2029.^7^ This would result in an increase of the mean base salary from $10 million in 2024,^4^ highlighting the importance for NBA franchises to keep players uninjured since the COR on the teams will greatly increase.

Johns et al^10^ performed a systematic review of the literature surrounding the career outlook and performance of professional athletes after Achilles tendon rupture and found that NBA players’ RTP rate was statistically significantly lower compared with that of other athletes in the NBA and the NFL with other orthopaedic injuries such as ankle fractures and ACL injuries. Additionally, the mean length of an NBA career of 4.8 years^16^ increases the stakes of an injury, where missing just 1 season could be up to 20% of a player's career. This highlights the need for a better evaluation of a player's longevity and value after an Achilles tendon rupture. Understanding these costs is useful when franchises look to alter their roster and manage the risk of long-term contracts.

### Limitations

Like other retrospective studies, this study has several limitations. Potential bias might be linked to the retrospective approach used in this analysis. Players with partial Achilles tendon tears were excluded, although the treatment protocols may overlap with those of complete tendon ruptures. The data were sourced from publicly available outlets, which may lack the accuracy of the cohort of a well-established database. Moreover, a more comprehensive database might have included specifics of surgical techniques, partial tears, and location of injury. The player categorization scale introduced in this paper has not been previously validated. A control group was not used in this study, which limits the generalizability of the study. Salary data may not be comprehensive, with incentives and bonuses missing. The calculated COR does not account for therapy-related costs such as physician visits and rehabilitation. Data on the type of surgery received (eg, internal brace, use of orthobiologics) and the specific tendon location of injury (proximal, distal, or midportion) for each player were not able to be obtained, which can affect the difficulty of the surgical reconstruction, rehabilitation, and potential outcome. These factors can affect recovery time and RTP and thus the COR, or the financial burden, for their respective franchises.

The number of games played during a player's season of injury and season after injury was used to calculate the number of games missed, which may have some limitations in accuracy. In a hypothetical example, a coach may choose not to play a reserve player immediately after the player's healed injury since he may have a smaller impact on team success than the All-Star players. This could affect statistics such as games missed, recovery time, and COR. Days missed also potentially suffers from the same limitation, but it still serves as a good metric of RTP.

## Conclusion

This study investigated the financial and performance implications of Achilles tendon ruptures among NBA athletes. Most players struggled to restore their preinjury performance, except for Reserve players. Our findings provide valuable insights into the complexities of COR and postinjury performance.
